# Using Sensor Technology to Measure Gait Capacity and Gait Performance in Rehabilitation Inpatients with Neurological Disorders

**DOI:** 10.3390/s22218387

**Published:** 2022-11-01

**Authors:** Maartje M. S. Hendriks, Marije Vos-van der Hulst, Ralf W. J. Weijs, Jaap H. van Lotringen, Alexander C. H. Geurts, Noel L. W. Keijsers

**Affiliations:** 1 Department of Research, Sint Maartenskliniek, Hengstdal 3, 6574 NA Nijmegen, The Netherlands; 2 Department of Rehabilitation, Donders Institute for Brain, Cognition and Behaviour, Radboud University Medical Center, 6500 HB Nijmegen, The Netherlands; 3 Department of Rehabilitation, Sint Maartenskliniek, 6574 NA Nijmegen, The Netherlands; 4 Department of Physiology, Radboud Institute for Health Sciences, Radboud University Medical Center, 6500 HB Nijmegen, The Netherlands; 5 Department of Rehabilitation, Basalt, 2543 SW Den Haag, The Netherlands; 6 Donders Institute for Brain, Cognition and Behaviour, Radboud University, 6500 GL Nijmegen, The Netherlands

**Keywords:** sensor technology, inertial measurement units, gait performance, gait capacity, physical activity, spontaneous gait characteristics, stroke, spinal cord injury, rehabilitation, gait

## Abstract

The aim of this study was to objectively assess and compare gait capacity and gait performance in rehabilitation inpatients with stroke or incomplete spinal cord injury (iSCI) using inertial measurement units (IMUs). We investigated how gait capacity (what someone can do) is related to gait performance (what someone does). Twenty-two inpatients (11 strokes, 11 iSCI) wore ankle positioned IMUs during the daytime to assess gait. Participants completed two circuits to assess gait capacity. These were videotaped to certify the validity of the IMU algorithm. Regression analyses were used to investigate if gait capacity was associated with gait performance (i.e., walking activity and spontaneous gait characteristics beyond therapy time). The ankle positioned IMUs validly assessed the number of steps, walking time, gait speed, and stride length (r ≥ 0.81). The walking activity was strongly (r ≥ 0.76) related to capacity-based gait speed. Maximum spontaneous gait speed and stride length were similar to gait capacity. However, the average spontaneous gait speed was half the capacity-based gait speed. Gait capacity can validly be assessed using IMUs and is strongly related to gait performance in rehabilitation inpatients with neurological disorders. Measuring gait performance with IMUs provides valuable additional information about walking activity and spontaneous gait characteristics to inform about functional recovery.

## 1. Introduction

Gait capacity is the most important determinant of independent living, social participation, and quality of life. It is often impaired in patients with neurological disorders such as stroke or spinal cord injury [1,2,3,4]. Therefore, regaining independent gait is a key objective in neurological patients [5,6,7], which is pursued from day one of their inpatient rehabilitation. It has been shown that early initiation of gait rehabilitation has beneficial effects on many physical and psychological aspects of functional recovery [8,9]. 

Hence, early initiated, task-specific, and sufficiently intensive gait training is an important principle in the rehabilitation of neurological patients aimed at the improvement of their mobility and health [10].

During inpatient rehabilitation, gait assessment is predominantly performed under standardized circumstances, such as planned therapy sessions or lab-based gait analyses [11]. This may result in a distorted ‘snapshot’ of patients’ abilities, as there is often a discrepancy between what patients are able to do during formal therapy time (referred to as ‘gait capacity’) and what they actually do beyond therapy time (referred to as ‘gait performance’) [12]. In other words, the assessment of gait capacity during formal therapy time is less ecologically valid than the assessment of gait performance during the remainder of the day [13]. It has been argued that objectively monitoring gait performance beyond therapy time might be of value to optimize individually tailored gait rehabilitation [14,15,16]. Previous studies focusing on rehabilitation inpatients mainly examined activity behavior [4,10,16] but did not report spontaneous gait characteristics, such as spontaneous gait speed or spontaneous stride length. Moreover, these studies did not distinguish between therapy time and time beyond therapy, even though it can be expected that monitoring walking and spontaneous gait characteristics beyond therapy time may provide valuable information to optimize individually tailored gait rehabilitation.

Inertial measurement units (IMUs) are able to record signals that allow inferences on both gait capacity and gait performance [17,18,19]. They are small, lightweight, body-worn, and user-friendly [20] and permit long-term gait recording in almost any environment [21]. In line with the above, previous studies in older adults found differences between gait assessed in standardized testing environments and gait performance in home settings [22,23]. Furthermore, they revealed that gait capacity testing might result in an overestimation of an individual’s gait performance [24]. However, currently, the greatest challenge is that most IMUs are not yet sufficiently validated for the assessment of pathological gait [25]. This is one of the reasons why gait capacity tests, such as the 10-m walk test (10mWT) or the 6-min walk test (6 MWT), are still a mainstay in clinical practice and studies monitoring gait performance are scarce. Yet, for inpatient rehabilitation, gait monitoring throughout the day holds promise to improve gait training and functional recovery by providing behavioral data that can be related to individual rehabilitation goals and progression [15,16]. 

The aim of this study was to objectively assess and compare gait capacity and gait performance in rehabilitation inpatients with stroke or incomplete spinal cord injury (iSCI) using IMUs. Gait capacity was operationalized as the gait speed recorded during the execution of a short walking circuit. For the assessment of gait performance, walking activity (i.e., number of steps and gait bouts) was distinguished from spontaneous gait characteristics (i.e., spontaneous gait speed and spontaneous stride length), both recorded beyond formal therapy time. Various steps were taken to compare gait capacity and gait performance, both assessed with IMUs. First, the validity of the IMU data for determining gait capacity was established by using video data as a reference. Second, gait performance (walking activity and spontaneous gait characteristics) was assessed. Third, the relationships between gait capacity and gait performance were determined. We hypothesized that the IMU data would be valid for assessing gait capacity in neurological patients. We further hypothesized that gait capacity would be strongly associated with the measures of gait performance. Furthermore, we expected that the maximum spontaneous gait speed would be similar to capacity-based gait speed, whereas the average spontaneous gait speed would be smaller than capacity-based gait speed. Evidence in support of our hypotheses would pave the way for IMU-based monitoring of gait capacity and gait performance in an objective, automated, and continuous way to inform about functional recovery in rehabilitation inpatients with neurological disorders.

## 2. Materials and Methods

### 2.1. Study Design and Approval

This observational cohort study was conducted at the rehabilitation center of the Sint Maartenskliniek (Nijmegen, The Netherlands). The study is part of the “Smarten the Clinic” project, which was approved by the regional medical ethics committee of Arnhem-Nijmegen (2018-4222).

### 2.2. Participants

Participants were hospitalized patients admitted to the rehabilitation center (both sexes, age ≥ 16 years) with either a first-ever ischemic or hemorrhagic stroke (Functional Ambulation Categories 2–5 [26]) or an incomplete Spinal Cord Injury (iSCI) (American Spinal Injury Association scale C/D/E [27]). Participants had to participate in formal gait training and not have any comorbidities that could affect their gait. Those who were unable to grant permission to participate due to language issues or cognitive impairments could not participate. Written and oral informed consent was obtained prior to participation. 

### 2.3. Equipment

Raw tri-axial accelerometer, gyroscope and magnetometer data were obtained using Shimmer^®^3 IMUs (Shimmer Sensing, Dublin, Ireland; 51 × 34 × 14 mm, 23.6 g) with a sampling frequency of 102.4 Hz and stored on a Microsoft^®^ Azure Platform. For a detailed description of the technological platform used, see Hendriks, et al. [28].

### 2.4. Gait Capacity Assessment

**Figure 1:**
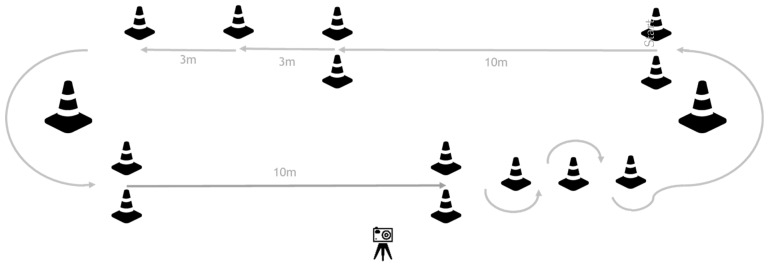
Gait capacity testing circuit. Participants were instructed to perform two laps of the circuit. In this way, they performed 12 subtasks during each circuit, including: four 10mWTs, four 3-m walks, two 180-degree curves, and two slaloms, including a 180-degree curve. After each subtask, participants had to stand still for at least 2 s before starting the next subtask.

During two gait training sessions on separate days, participants completed a pre-specified circuit (two laps) at their own comfortable speed to assess their gait capacity (Figure 1). During each circuit, 12 subtasks were performed: four ten-meter walk tests (10mWTs), four 3-m walks, two 180-degree curves, and two slaloms, including a 180-degree curve. Participants were instructed to stop walking for at least two seconds after each subtask to be able to analyze each subtask as a separate gait bout. The circuit was videotaped to obtain visual reference data for validity testing of the gait parameters calculated by the IMU algorithm: a number of steps and walking time for all subtasks, gait speed, and stride length for the 10mWTs. 

### 2.5. Gait Performance Assessment

To monitor gait performance, all participants were instructed to wear two IMUs strapped cranially to the lateral malleoli of the left and right ankle during the daytime of five to eight consecutive measurement days, referred to as one measurement period. Only weekdays on which data were actually obtained were included in the analyses. From these data, gait bouts recorded during the gait capacity test circuits were excluded. Within the measurement period, participants followed their regular inpatient rehabilitation program. For the assessment of gait performance, walking activity (i.e., number of steps and gait bouts) was distinguished from spontaneous gait characteristics (i.e., spontaneous gait speed and spontaneous stride length), both recorded beyond formal therapy time. Therapy time was labeled based on individual patient planning records. All gait bouts during any type of formal therapy were excluded. 

### 2.6. Data Processing and Analysis

#### 2.6.1. IMU Gait Algorithm

Data from the ankle-positioned IMU were analyzed based on the gait algorithm of Patterson, et al. [29]. These analyses yielded data for each gait bout. At least two seconds of gait pause were used to determine the end of a gait bout and the start of the next. Vertical gyroscopic data were used to extract mid-stance points, which were used to detect strides and stride time. Accelerometer data between mid-stance points were used to calculate stride length. For each stride, stride velocity was calculated based on the stride length divided by the stride time. Subsequently, the mean velocities across the left and right strides of a gait bout were used to define the velocity per bout. The total number of steps of a gait bout was defined as the sum of the number of left-and right-sided strides. 

#### 2.6.2. Gait Capacity

The circuits (Figure 1) of each participant were video recorded to determine the capacity-based number of steps and walking time for each correctly executed subtask. If participants did not stop for at least two seconds, the last subtask and the subsequent one was excluded. For each correctly executed 10mWT, capacity-based walking time was used to calculate capacity-based gait speed (10 m/capacity-based walking time), and the capacity-based number of steps was used to calculate capacity-based stride length (10 m/(capacity-based number of steps × 0.5)). A 10mWT was considered correct if the participant started walking at the start line, continued walking 10 m without a pause, and stopped at the finish line. 

#### 2.6.3. Gait Performance

The gait algorithm validation revealed that sometimes (see results), stride detection of one of the two sensors failed. In these cases, the gait speed, and the total number of steps were based on just one sensor, the latter by doubling the number of strides detected by this sensor. Gait bouts were included in the analysis if they consisted of at least 3 steps and the IMU was in an upright position indicated by a mean accelerometer value of the longitudinal sensor of at least (−)8 m/s^2^. 

For the assessment of gait performance, we distinguished between walking activity and spontaneous gait characteristics. Walking activity parameters were the number of bouts, the number of steps, the distance covered, and walking time per hour. In addition, the mean and maximum number of steps, distance covered, and walking time across gait bouts were calculated. Spontaneous gait characteristics were based on gait bouts comprising 10 to 100 steps. Spontaneous gait parameters were average and maximum spontaneous gait speed and spontaneous stride length. The average of the five bouts with the 2nd to 6th highest spontaneous gait speed was used to define the maximum spontaneous gait speed and maximum spontaneous stride length. Detailed analyses revealed that the algorithm sometimes defined two or more strides as one single stride resulting in a disproportionally increased stride length. Therefore, bouts with stride lengths larger than 1.33 times the median of the capacity-based stride length were excluded from the analysis. 

Table 1 provides an overview of the used terminology and associated outcome parameters.

### 2.7. Statistical Analysis

All descriptive statistics were presented as mean ± SD if normally distributed or median (min–max) if not normally distributed. The Shapiro–Wilk test was used to determine normality. 

#### 2.7.1. Validation of IMU Data and Algorithm

Descriptive statistics were used for both video- and IMU-based gait capacity parameters. Average capacity-based gait speed and stride length were calculated as the mean of all individual means of correctly performed 10mWTs. To test the validity of the ankle-positioned IMU data and gait algorithm, univariate linear regression analysis was performed for the following parameters obtained during the execution of the gait circuits: capacity-based number of steps and walking time for each subtask and capacity-based gait speed and stride length for the 10mWT. In line with the literature [30,31], correlation coefficients (r) > 0.75 were considered sufficient to support the validity of the IMU gait algorithm. 

#### 2.7.2. Gait Performance

Descriptive statistics were used for all gait performance parameters. Walking activity measures were calculated as the median of all individual walking activity outcome measures in which the total walking activity was divided by the total number of bouts and/or total hours measured. Maximum walking activity measures were calculated as the median of all individual maximum walking activity outcomes. The number of participants reaching a covered distance of 50, 100, and 300 m and achieving a gait speed of 0.4, 0.8, and 1.2 m/s was determined for walking activity beyond therapy time. Average and maximum spontaneous gait speed and stride length were calculated as the mean of all individual means for all included gait bouts (described in the previous section). 

#### 2.7.3. Relationship between Gait Capacity and Gait Performance

Univariate linear regression analyses were performed to test whether capacity-based gait speed was related to the walking activity (i.e., number of bouts/hour, number of steps/hour, walking time, and distance covered). Wilcoxon signed-rank tests were used to compare spontaneous gait characteristics (i.e., spontaneous gait speed and spontaneous stride length) to capacity-based gait speed and stride length. In addition, univariate linear regression analyses were used to test whether capacity-based gait characteristics were related to spontaneous gait characteristics.

As a rule of thumb [31], a correlation coefficient (r) > 0.75 was considered to indicate a strong relationship. Slope (β) and intercept (a) were provided for univariate linear regression analysis between capacity-based gait characteristics and spontaneous gait characteristics. *p*-values < 0.05 were considered significant. To adjust for multiple comparisons between gait capacity and performance (six in total; four for walking activity and two for spontaneous gait characteristics), a Bonferroni correction was applied (*p* < 0.0083).

Data analysis and statistical testing were performed using MATLAB (R2017b, Version 9.3.0.713579, The MathWorks Inc., Natick, MA, USA) and RStudio (RStudio Team (2020). RStudio: Integrated Development for R. Version 3.6.3. RStudio, PBC, Boston, MA, USA).

## 3. Results

### 3.1. Participants

**Figure 2:**
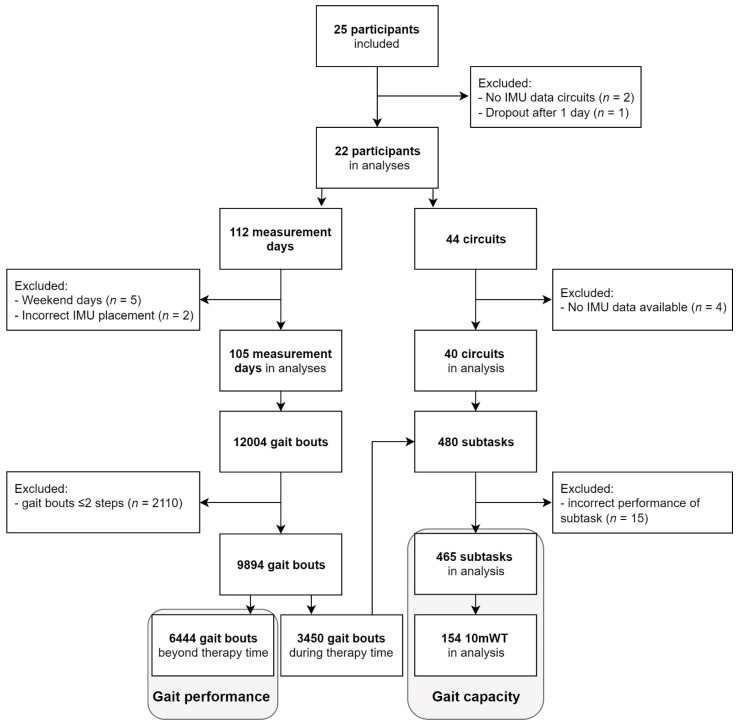
Flowchart of participants, measurement days, circuits, and gait bouts. Gait performance analysis was based on 6444 gait bouts performed beyond therapy time. Gait capacity analysis was based on 465 subtasks performed during circuits in therapy time, including 154 10mWTs.

Twenty-two participants (aged 66 ± 12 years, Table 2) were included, comprising 11 first-ever strokes (aged 69 ± 9 years) and 11 iSCI patients (aged 63 ± 13 years). In 105 days (9.7 ± 1.2 h/day), a total of 9894 gait bouts of at least three steps were performed during 249 h (24%) of therapy time and 768 h (76%) beyond therapy time. In 1565 of 9894 gait bouts (16%), stride detection was lacking from one sensor, but these bouts could still be used for analysis based on data from the other sensor. A total of 6444 gait bouts were performed beyond therapy time and used in the gait performance analysis. For analysis of gait capacity, 465 subtasks comprising 154 10mWTs were included. See Flowchart (Figure 2) for details regarding the number of participants, measurement days, circuits, and gait bouts.

### 3.2. Validation of IMU Data and Algorithm

**Figure 3:**
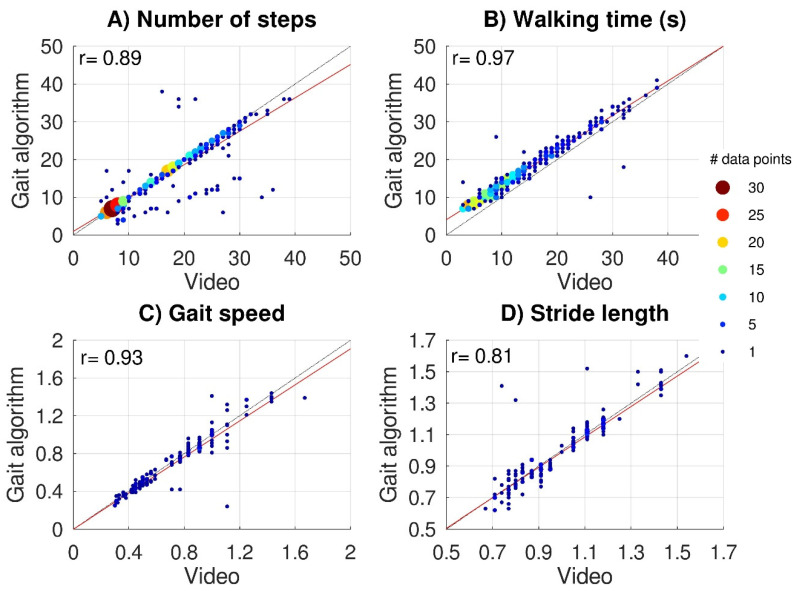
Validation of IMU data. Scatter plots of video-based versus IMU-based gait parameters. The upper panels show the capacity-based number of steps (**A**) and capacity-based walking time (**B**) for 465 subtasks performed by 22 subjects. Lower panels show capacity-based gait speed (**C**) and capacity-based stride length (**D**) for 154 10mWTs. Black dashed lines represent perfect-fit (x = y), whereas the red lines represent the regression lines. Multiple (overlapping) data points are indicated with bigger color-coded data points.

Average values and standard deviations across subjects for the 10mWTs were 0.73 ± 0.30 m/s for capacity-based gait speed and 0.99 ± 0.21 m for capacity-based stride length. Figure 3 shows scatter plots of video-based versus IMU-based data for the capacity-based number of steps (Figure 3A) and capacity-based walking time (Figure 3B) (all subtasks) and for capacity-based gait speed (Figure 3C) and capacity-based stride length (Figure 3D) (10mWT). The video-based data all significantly correlated with the IMU-based data: capacity-based number of steps *r* = 0.89, *p* < 0.001, capacity-based walking time *r* = 0.97, *p* < 0.001, capacity-based gait speed *r* = 0.93, *p* < 0.001, and capacity-based stride length *r* = 0.80, *p* < 0.001. For 48 subtasks (10%), the gait algorithm over-(*n* = 10) or underestimated (*n* = 38) the capacity-based number of steps by more than 25%. The lack of step detection on one side caused an underestimation in 32 subtasks. Although the gait algorithm was responsible for most outliers, some outliers (6/10) were caused by swapping the left and right sensors by the participant, which we considered a human interaction error (Hendriks, et al., 2020).

### 3.3. Gait Performance

A total of 6444 gait bouts comprising 206,599 steps (60%) were performed beyond therapy time. Table 3 shows the corresponding walking activity and spontaneous gait characteristics. Beyond therapy time, 17, 13, and 4 participants covered a walking distance of 50, 100, and 300 m, respectively. All participants, except two, performed gait bouts between 10 and 100 steps. The numbers of participants reaching a spontaneous gait speed of at least 0.4, 0.8, and 1.2 m/s were 21, 14, and 4, respectively.

### 3.4. Relationship between Gait Capacity and Gait Performance

**Figure 4:**
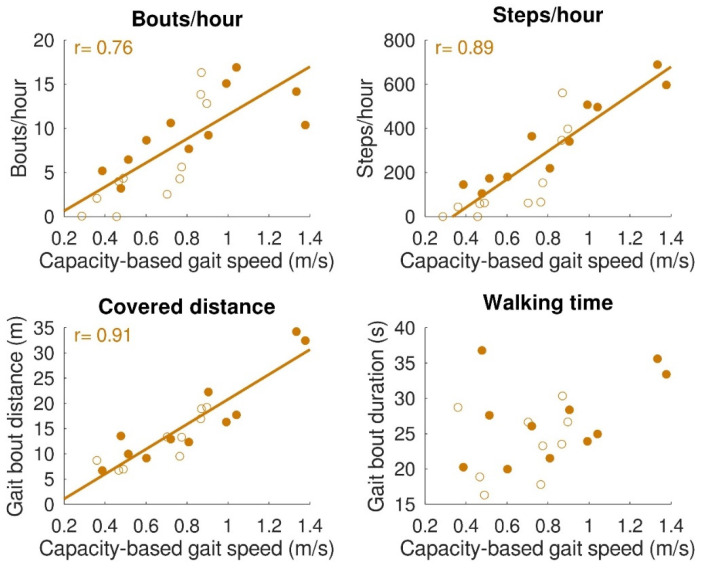
Relation between gait capacity and gait performance (walking activity). Bouts/hour (top left), steps/hour (top right), and distance covered (bottom left) were significantly correlated with capacity-based gait speed. Walking time (bottom right) was not significantly correlated with capacity-based gait speed. Filled dots indicate individual stroke patients, and open dots indicate individual patients with iSCI. In the case of a significant correlation, regression lines are depicted.

**Figure 5:**
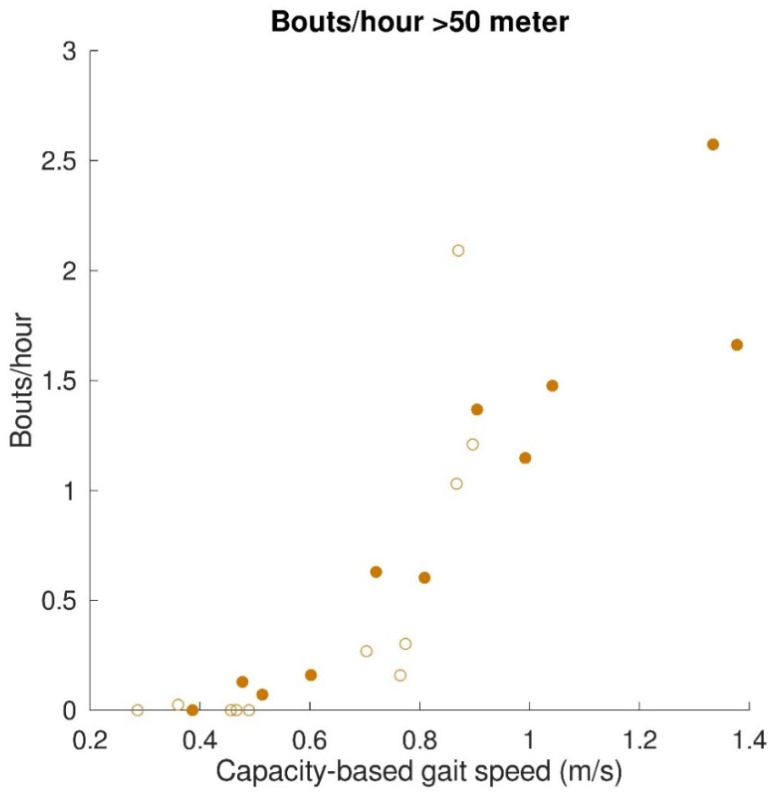
Relation between gait capacity and gait performance (walking activity; distance covered > 50 m). The mean number of bouts/hour with a distance covered > 50 m are represented by filled dots for stroke patients and by open dots for patients with iSCI.

The relationships between gait capacity and walking activity parameters are shown in Figure 4. Capacity-based gait speed was significantly (positively) correlated with the number of bouts/hour (*r* = 0.76, *p* < 0.001), number of steps/hour (*r* = 0.89, *p* < 0.001), and distance covered per gait bout (*r* = 0.91, *p* < 0.001). Capacity-based gait speed was not significantly correlated with walking time per gait bout (*r* = 0.42, *p* = 0.06). Furthermore, a relationship was found between capacity-based gait speed and walking distance above 50 m, which is depicted in Figure 5. All subjects with a capacity-based gait speed above 0.85 m/s had at least one gait bout per hour of more than 50 m.

**Figure 6:**
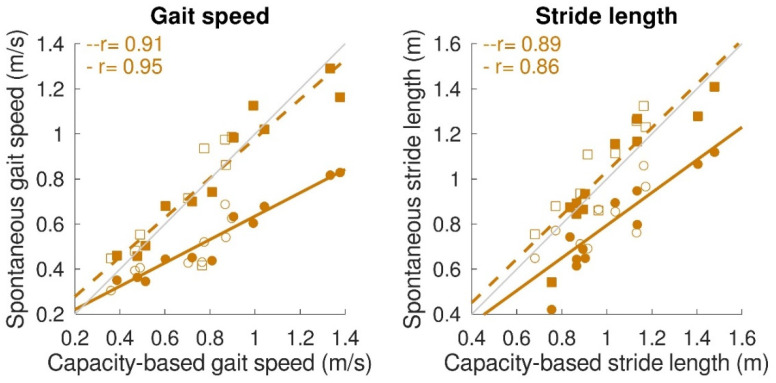
Relation between gait capacity and gait performance (spontaneous gait characteristics). Maximum spontaneous and average spontaneous gait speed (**left panel**) and maximum spontaneous and average spontaneous stride length (**right panel**) were significantly correlated with capacity-based gait speed and capacity-based stride length, respectively. Circular dots with solid regression lines represent the average spontaneous gait characteristic, while square dots with the dashed regression lines represent the maximum spontaneous gait characteristic. A single participant is represented by a filled dot for stroke patients and an open dot for iSCI patients. Grey lines represent perfect fit (x = y), whereas the orange lines represent the regression lines.

Concerning the spontaneous gait characteristics, the average spontaneous gait speed (0.51 ± 0.16 m/s) was significantly lower (*p* < 0.001) than the capacity-based gait speed (0.73 ± 0.30 m/s). However, maximum spontaneous gait speed (0.78 ± 0.27 m/s), maximum spontaneous stride length (1.04 ± 0.23 m), and average spontaneous stride length (0.92 ± 0.18 m) were not significantly different from the capacity-based measures. Figure 6 shows the relation between capacity-based gait characteristics and gait performance. Average spontaneous gait speed and stride length were significantly correlated with capacity-based average gait speed (average spontaneous gait speed: *r* = 0.95, *p* < 0.001; β = 0.52, a = 0.12; average spontaneous stride length: *r* = 0.86, *p* < 0.001; β = 0.73, a = 0.07). Maximum spontaneous gait speed and maximum spontaneous stride length were significantly correlated with capacity-based gait speed and stride length. The slope for both variables was close to 1 (maximum spontaneous gait speed: *r* = 0.91, *p* < 0.001, β = 0.88, a = 0.10; maximum spontaneous stride length: *r* = 0.89, *p* < 0.001, β = 0.97, a = 0.06).

## 4. Discussion

This study aimed to objectively assess and compare gait capacity and gait performance in rehabilitation inpatients with stroke or iSCI using IMUs. The IMU-based gait algorithm was found to be valid for gait assessment. As hypothesized, gait capacity was strongly related to gait performance. The walking activity beyond therapy time was strongly and positively related to capacity-based gait speed. For spontaneous gait characteristics, maximum spontaneous gait speed and stride length were similar to capacity-based gait speed and stride length, respectively. Congruent with our hypothesis, the average spontaneous gait speed was half the capacity-based gait speed. 

Because measuring pathological gait with IMUs in daily life remains challenging [25,32], our first aim was to validate our IMU-based gait algorithm in rehabilitation inpatients with stroke or iSCI. The ankle-positioned IMUs were valid for the assessment of the capacity-based number of steps, walking time, gait speed, and stride length when tested during a standardized measurement protocol, including turns, slaloms, and short walking bouts. The validity of the IMU-based assessment of capacity-based gait speed, stride length, and number of steps during regular walking is in line with previous studies using ankle-worn sensors [29,33,34,35]. A limitation of the algorithm was that one sensor sometimes missed stride detection, which occurred in 8% during standardized testing and in 16% beyond standardized testing. Additional detailed analyses showed that most missing strides during gait capacity tests (32/38 gait bouts) occurred in participants with a gait speed lower than 0.5 m/s, irrespective of the patient group. Furthermore, we visually checked the IMU data randomly for non-detected gait bouts beyond therapy time but did not find any gait bouts that were missed by both IMUs. Therefore, we are confident that all gait bouts and their corresponding number of steps were correctly detected. Previously, we have shown that a sensor-based technological platform comprising IMUs can be used continuously and in an automated way in a clinical rehabilitation setting [28]. Taken together, we conclude that gait performance can be measured objectively, continuously, and automatically by using sensor technology during inpatient rehabilitation.

Rehabilitation inpatients performed a substantial part of the walking activity (76%) beyond therapy time in which they performed more than half of their total gait bouts and number of steps (65% and 60%, respectively). The current study’s median of 177 steps/hour beyond therapy time is in the lower range. People living with disability and/or chronic illness show, on average, 100–733 steps/hour based on 1200–8800 steps/day [23]. However, our findings correspond to stroke patients with a daily number of steps of 2922 steps/day (approximately 250 steps/hour) two months post-stroke [36] and to ambulatory spinal cord injury patients taking 1097 steps/day (approximately 90 steps/hour) [16]. Interestingly, the number of steps per day two months post-stroke was predictive of the number of steps per day one-year post-stroke [36]. Hence, it seems valuable to monitor gait performance beyond therapy time during inpatient rehabilitation, as patients are fairly active beyond therapy time, and walking activity has clear predictive value. 

Congruent to our hypothesis, gait capacity was strongly and positively related to the walking activity. This is in accordance with a previous study showing that stroke patients with a gait speed below 0.8 m/s perform less moderate to vigorous physical activity than stroke patients with a gait speed above 0.8 m/s [37]. The only walking time per gait bout was not significantly correlated with capacity-based gait speed. Due to a low gait speed, the total walking time of short gait bouts is comparable to the prolonged gait bouts of participants with higher gait speed. Nevertheless, the strong correlations found between capacity-based gait speed and steps/hour and distance covered emphasize the importance of gait speed being an indicator of walking activity. This is also reflected by the relation between capacity-based gait speed and the number of gait bouts with a distance above 50 m. Participants with a gait speed below 0.85 m/s had less than one gait bout per hour above 50 m, whereas participants with a gait speed above 0.85 m/s showed one to three of these gait bouts per hour. For people with a lower-limb prosthesis after a leg amputation, a distance covered of 50 m is used as a minimum requirement for indoor and outdoor walking [38,39]. It is, therefore, a meaningful indicator of independent living. A gait speed of about 0.8 m/s seems to be a turning point in walking independence. This finding supported the walking velocity categories used by Fini, et al. [37] and described by Perry, et al. [40], in which unlimited community ambulators had a gait speed above 0.8 m/s and limited community ambulators a gait speed below 0.8 m/s. Sensor technology provides the opportunity to measure gait performance, such as covered distance during the day, which can be used as an indicator of independent living after inpatient rehabilitation discharge.

Capacity-based gait characteristics were also strongly related to spontaneous gait characteristics. The average spontaneous gait speed of 0.51 m/s and stride length of 0.92 m was in the expected range for stroke and iSCI patients, as presented in the literature [41,42,43]. Furthermore, average spontaneous gait speed and stride length were strongly correlated with the respective gait-capacity measures. However, for average spontaneous gait speed, it appeared to be half of the gait speed reached during the capacity test (indicated by a slope of 0.5). Previous studies in healthy community-dwelling older adults also found that daily life gait speed is lower than during standardized walking tests [24,44]. In the current study, we focused on the maximum spontaneous gait characteristics as well. It became clear that both maximum spontaneous gait speed and stride length were similar to capacity-based gait measures, which endorses the possibility of estimating gait capacity based on gait performance measurements beyond therapy time. This could save sparse standardized testing time and manual data entry time, which would benefit training and coaching. 

Despite the valuable findings, we are aware of some study limitations. The wearing time of the IMUs was different between participants due to various personal and/or practical reasons. Therefore, we chose to report the number of gait bouts and walking activity per hour instead of per day. We do not think this has affected our results, as it is a good reflection of clinical practice. Furthermore, therapy time was based on the patient’s therapy schedules. However, possible (slight) deviations from actual therapy time were not registered for reasons of feasibility and privacy. Although the IMU-based gait algorithms have shown to be valid [25,34,35], it still remains challenging to validly measure impaired gait. We, therefore, validated the gait algorithm during the gait capacity assessment. We are aware of some outliers, which indicates that the algorithm can still be improved. Recently, several new sophisticated gait algorithms have been published, including multi-model and sensor fusion, which combine multiple algorithms and/or sensors to provide a more extensive gait analysis comprising different environments [45], as well as approaches based on deep learning [46]. Important to note is that for clinical use, the gait algorithms have to be accurate enough to detect the minimal clinically important difference (MICD), which is between 0.1–0.2 m/s for gait speed in various rehabilitation patient groups [47]. Although the gait algorithm was responsible for most outliers, some outliers were caused by swapping the left and right sensors by the participant, which we considered a human interaction error [28]. For the successful implementation of sensor technology in the clinical setting, it is important to improve the gait algorithm for gait speeds below 0.5 m/s and to reduce the number of human interaction errors to a minimum.

In summary, gait capacity can be validly assessed using IMUs in neurological patients with impaired gait during inpatient rehabilitation. Gait capacity and gait performance were strongly related, but gait performance measures provided valuable additional information about walking activity and spontaneous gait characteristics. Using IMU technology beyond therapy time, therapists may fully dedicate their sparse therapy time to training and coaching. Lastly, continuous monitoring using IMUs during inpatient rehabilitation could be a stepping stone toward gait monitoring at home. Using sensor technology to monitor gait capacity and gait performance in a person’s natural environment will improve opportunities to pursue goals, evaluate patient progression, and provide personalized and optimized rehabilitation treatment.

## Figures and Tables

**Table 1:** Overview of used terminology and associated outcome parameters.

Terminology	Explanation	Outcome Parameters
**Gait capacity**	A person’s ability to walk in a controlled and safe environment. It reflects what someone can do (in this study during formal gait therapy).	Capacity-based number of steps Capacity-based walking time Capacity-based gait speed Capacity-based stride length
**Gait performance**	A person’s actual execution of walking activity in an uncontrolled and unsupervised environment. It reflects what someone actually does (in this study, beyond therapy time).	See qualifiers (below)
*Walking activity*	A qualifier of gait performance: a person’s actual walking activities expressed per hour, based on gait bouts (continuous periods of walking).	Number of gait bouts/hour Number of steps/hour Distance covered/hour Walking time/hour Mean and maximum: ○ Number of steps/bout ○ Distance covered/bout ○ Walking time/bout
*Spontaneous gait characteristics*	A qualifier of gait performance: a person’s actual gait speed and stride length during bouts of spontaneous walking activity.	Average spontaneous gait speed Maximum spontaneous gait speed Average spontaneous stride length Maximum spontaneous stride length

**Table 2:** Individual demographic and clinical characteristics of the participants.

Disease		Sex (M/F)	Age (Years)	Affected Hemisphere (L/R)	FAC Score (0–5)	AIS Score	Lesion Level
Stroke (*n* = 11)	1	F	77	R	2	-	-
	2	M	58	L	5	-	-
	3	F	79	L	4	-	-
	4	M	50	R	5	-	-
	5	F	73	R	4	-	-
	6	F	66	L	4	-	-
	7	M	67	L	4	-	-
	8	F	81	L	4	-	-
	9	M	74	R	2	-	-
	10	M	63	R	3	-	-
	11	M	66	L	4	-	-
iSCI (*n* = 11)	12	M	48	-	-	C	C3
	13	M	53	-	-	D	L3
	14	M	76	-	-	D	T11
	15	M	80	-	-	D	L2
	16	M	78	-	-	D	T3
	17	M	38	-	-	D	C4
	18	F	63	-	-	D	T4
	19	F	62	-	-	D	T9
	20	M	68	-	-	D	C4
	21	F	55	-	-	D	T11
	22	M	68	-	-	D	C2

**Table 3:** Gait performance (beyond therapy time): walking activity and spontaneous gait characteristics.

Walking Activity (bouts > 2 Steps)	Median (Range)
Steps/hour	177 (0–689)
Bouts/hour	7 (0–17)
Distance covered/hour (m)	75 (0–482)
Walking time/hour (s)	169 (0–505)
Steps/bout	28 (0–58)
Maximum steps/bout	280 (0–2165)
Distance covered/bout (m)	13 (0–34)
Maximum distance covered/bout (m)	137 (0–2169)
Walking time/bout (s)	24 (0–37)
Maximum walking time/bout (s)	205 (0–923)
**Spontaneous gait characteristics (bouts 10–100 steps)**	**Mean (SD)**
Average spontaneous gait speed (m/s)	0.51 ± 0.16
Maximum spontaneous gait speed (m/s)	0.78 ± 0.27
Average spontaneous stride length (m)	0.92 ± 0.18
Maximum spontaneous stride length (m)	1.04 ± 0.23

## Data Availability

The data presented in this study are available upon reasonable request from the corresponding author.
